# Scale development of apparel customization brand value: From the perspectives of practitioners and consumers

**DOI:** 10.3389/fpsyg.2022.933224

**Published:** 2022-08-10

**Authors:** Hao Li, Li-Wen Gu, Xiao-Gang Liu, Yan-Wen Ruan

**Affiliations:** ^1^College of Fashion and Design, Donghua University, Shanghai, China; ^2^Shanghai International College of Fashion and Innovation, Donghua University, Shanghai, China; ^3^School of Textile and Fashion, Shanghai University of Engineering Science, Shanghai, China

**Keywords:** China apparel customization brands, brand value, customization practitioners, consumers, scale development, brand value dimensions

## Abstract

China apparel customization brands (CACBs) have been recently growing in massive quantities despite being in their infancy stages of brand value building. Although scholars have proven brand value’s importance in sustainable brand growth, studies on the specific context of CACBs are still limited. This research proposes a conceptual framework of CACBs’ brand value measured dimension based on previous studies and divides brand value into both general and specific dimensions. Accordingly, qualitative (semi-structured interviews) and quantitative (online survey) studies were conducted from the perspectives of practitioners and consumers. Ultimately, a scale of 30 items with nine dimensions was generated. Results reveal that brand association in the general dimensions and brand service in the special dimensions were the largest promoters of CACB brand value. Hence, practitioners should pay more attention to dimensions of cognitive conflicts. Practical suggestions for apparel customization marketers are proposed to build and enhance brand value.

## Introduction

Recently, the rapid development of China apparel customization brands (CACBs) has pushed them to be more service-oriented, fashionable, digital, and multi-category (Liu et al., 2020; Lang et al., 2021; Kliestik et al., 2022). With this, CACBs face severe challenges such as inconspicuous brand characteristics, weak product innovation, high costs of consumer acquisition, and low consumer engagement (Tangchaiburana and Techametheekul, 2017; Park and Yoo, 2018). Additionally, complex customization processes, unreasonable prices, and low-quality after-sales services disincentivize consumers (Merle et al., 2010; Liu et al., 2020), but can be solved by identifying dimensions that construct brand value for brand building (Sok and O’Cass, 2011; Saleh and Alotaibi, 2018; Cambra-Fierro et al., 2021). Brand value is the sale or replacement price of a brand (Raggio and Leone, 2009), and it represents the extent to which a brand increases or decreases the total value of a firm (Yeung and Ramasamy, 2008). Brand value dimensions are market-driven, reflecting the changes in brand value (Aaker, 1996; Guha Roy et al., 2022). Hence, identifying the problems faced by brands could provide targeted suggestions for brand building (Çifci et al., 2016; Debnath et al., 2016; Su, 2016; Chi et al., 2020; Guha Roy et al., 2022). This study focuses on the problems faced by customization brands as an entry point, constructed a brand value measurement scale suitable for CACB, and provided decision-making suggestions for the CACBs’ development.

Previous studies have typically investigated brand value dimensions either from consumers’ perspective or from the corporate perspective (Merle et al., 2010; Thirumalai and Sinha, 2011; Liu and Wang, 2012; Yoo and Park, 2016), these studies skew the current literature on brand value ungeneralizable (Jones, 2005). This study’s multi-perspective research is therefore conducive to better understanding brand value; it provides a measurement for future researchers to study the brand value of CACBs by expanding the measurement dimension of brand value using both consumer and corporate perspectives following to the characteristics of customization apparel brands. Further, this study discusses the significance of each brand value measurement dimension for CACBs, providing brand operators with more specific value enhancement strategies. The aims of this study are threefold: First, it aims to identify the brand value dimensions of CACBs. Next, it seeks to develop a scale measuring those dimensions. Lastly, it provides a decision-making basis and framework for CACBs to enhance brand value.

## Literature review

### Apparel customization brand

Customization is the action of making or changing something according to the needs of the buyers or users (Lang et al., 2021). Apparel customization refers to clothing production created with the consumer’s participation according to their fit, specifications, design or the combination of the above factors (Senanayake Muditha and Little Trevor, 2010; Seo and Lang, 2019). Apparel customization benefits consumers by providing unique products that meet individual needs and providing a hedonic experience during the shopping process (Cho and Fiorito, 2009).

Due to market digitalization and the apparent surge in personalized demand, CACBs have generally shown low prices and Internet-based characteristics. Traditional handmade custom brands have been actively trying to use information technology to transform their business models. Tailors and salespeople in particular are able to recommend clothing to consumers that meet their specific needs based on a style intelligent recommendation system (Hao et al., 2020).

Therefore, customization brands enhance brand value by providing personalized products, convenient services and comfortable customization experiences. They are required to ensure product quality and improve their intelligence and information system to realize personalized products (Liu et al., 2020). Fast and convenient service is the basic attribute of customization brands, one that requires them to increase service ability (Sok and O’Cass, 2011). Consumers are involved in the product customization process, which positively drives their emotional attachment to the product and consequently optimize their customization experience (Lang et al., 2021).

### Consumer-based brand value

Brand value is largely considered as the financial value of a brand from the firm’s perspective (Isberg and Pitta, 2013; Laghi et al., 2020). However, brand valuation should include both financial and consumer behavior information (Heberden, 2011). Mixed methods of brand value evaluation from both financial and consumer perspectives have also been recently used (Alcaide et al., 2021). Hence, consumer factors are essential for building and improving brand value.

In previous work, consumer-based brand value started from the cognition of consumers about a familiar brand, which has become a dominant perspective when researching brands among academics and practitioners (Yoo and Donthu, 2001). Keller (1993) proposed the consumer-based brand equity model and defined it as the differential effect of consumers’ brand marketing responses which were based on brand knowledge. Similarly, Aaker (1996) proposed the “brand equity ten” model with consumers’ perception of the brand in four dimensions——loyalty, perceived quality, association, and awareness. Most studies measured consumer-based brand value from perceived quality, associations, brand image, loyalty, trust, consumer satisfaction, brand meaning, and brand sympathy (Keller, 1993; Yoo and Donthu, 2001; Burmann et al., 2009; Winzar et al., 2018; Chi et al., 2020; Cambra-Fierro et al., 2021; Zia et al., 2021; Guha Roy et al., 2022; Kliestik et al., 2022; Yuwono and Anandya, 2022).

Providing consumers with personalized products and services is the nature of customization brands, which leads to consumers judging the overall performance of the brand from the aspects of perceived product quality, brand image and brand experience (Zeithaml, 1988; Keller, 1993; Sharp, 1996; Ahmad et al., 2021). These aspects are important for a customization brand, requiring the brand to improve its ability of brand service and product fabrication to enhance satisfaction. However, existing research on consumer-based brand value rarely considers the perspective of brand service and product fabrication together, thus causing current customization brands with service-orientation attributes to lack a comprehensive brand value measurement criterion.

### Brand value dimensions of China apparel customization brand

Although most scholars agree on the measures of consumer-based brand value (Frías-Jamilena et al., 2018; Yang et al., 2019; Cho and Hwang, 2020; Chokpitakkul and Anantachart, 2020; Cambra-Fierro et al., 2021; Pina and Dias, 2021), these measures are scattered due to the recurring of the components forming brand value dimensions across different brand types. According to the research object of brand value, scholars have examined the measures of brand value from different perspectives, such as grouping by global and local brands (Zarantonello et al., 2020), the nature of fast fashion (Su, 2016), destination brands (Tasci, 2018), etc. China apparel customization brands (CACBs) refer to the brands operating in China that adopt the made-to-order method for consumers to produce clothing. As an important component of Chinese fashion brands, CACBs can be divided into three types (Liu, 2013), all of which are included in this study: high-end customization fashion brands; customization production line sub-brand; mass customization fashion brand. Some of the brand value dimensions universally used in fashion brand value evaluation are also applicable for apparel customization brands (Calvo Dopico and Calvo Porral, 2012), hence their need to be included herein. Meanwhile, because of the uniqueness of apparel customization brands compared to mass-production brands, it is necessary to conduct specific research on the brand value dimensions where the apparel customization brands show particularity. Hence, brand value dimensions are divided herein into either general or special dimensions based on the characteristics of customization apparel brand value to ensure the sufficient quantity and quality of the measurement dimension.

#### General dimensions

General dimensions refer to the dimensions that are applicable to most fashion brands and have been verified (Aaker, 1996). A set of examined general dimensions can provide guidance and structure for any customization brand, including perceived product quality, brand image, brand association, self-brand connection and brand culture (Zeithaml, 1988; Keller, 1993; Aaker, 1996; Steenkamp et al., 2003; Maden, 2013; Cho et al., 2015; Ye et al., 2015; van der Westhuizen, 2018; Sarkar et al., 2021). Brand image and brand association are the most intertwined dimensions (Cho and Fiore Ann, 2015). Thus, this study adopts the definition of brand association proposed by the research of Keller (1993). Below is the individual definition for each of the five general dimensions mentioned above.

##### Brand association

Brand association refers to anything related to the brand in memory (Aaker, 1996), including attributes, benefits, and attitudes (Keller, 1993). Brand association will be stronger when it is based on many experiences or exposures (Crawford Camiciottoli et al., 2014), thus reflecting the importance of customization brands to establish connections with consumers.

##### Brand image

Brand image is defined as perceptions about a brand reflected by the brand association in the consumer recall. Brand image depends on the degree of brand association, reflecting its favorability, strength, and uniqueness. It, therefore, is the amalgam of brand association (Keller, 1993; Cho et al., 2015; Kim and Oh, 2020). A customization brand image is built by connecting the nodes in consumer memory with the brand nodes to distinguish brand meaning for consumers (Cho et al., 2015).

##### Perceived product quality

Perceived product quality refers to consumers’ subjective judgment of product quality (Gök et al., 2019; Rosillo-Díaz et al., 2020). Consumers judge whether the quality of a product meets their expectations based on their own perception of the product’s materials, functions, performance, and manufacturing techniques (Bovea and Pérez-Belis, 2012; Smith et al., 2012). Perceived product quality can significantly impact brand value (Gill and Dawra, 2010), hence, related research is conducive to enhancing brand value.

##### Self-brand connection

Self-brand connection refers to the linking degree between consumers’ self-concept and the brand and reflects the consumer-brand relationship (Escalas and Bettman, 2003; van der Westhuizen, 2018). Self-concept refers to the way consumers think and feel about who and what they are (van der Westhuizen, 2018). For example, a person who perceives themself as a business elite would purchase products from a brand of customized clothing that they believe the business elite wear because the brand can express who the person is the brand connects to the consumer’s self-concept. Strengthening the relationship between brand and self-concept helps improve self-brand connection, thereby increasing brand value (Escalas and Bettman, 2003; Ye et al., 2015). For customization brands, the relationship can be fueled through products designed to represent consumers’ self-images and are subsequently expressed to others (Escalas and Bettman, 2003; Saleh and Alotaibi, 2018; Lang et al., 2021; Sarkar et al., 2021).

##### Brand culture

Brand culture refers to the combination of elements (e.g., names, nouns, logos, symbols, designs) that identify and distinguish products or services as well as the historical and cultural traits accumulated in the combination of these elements and the cultural phenomena represented in its operation and services (Yang, 2010; Maden, 2013). Culture has a significant impact on individual behavior and way of thinking, affecting the personal decision making as well as brand perception of consumers (Lam, 2007). Because brand culture is the source of brand value, conversely, a brand without cultural nourishment lacks vitality (Yang, 2010; Purani and Jeesha, 2021). Shaping brand culture needs to consider its own history and essence accumulated in its long-term development, providing the basis for its brand value strategy (Yang, 2010; Yan, 2011; Schroeder et al., 2017). Brand culture is an important element for customization brands, attracting consumers consistent with the brand’s value or philosophy (Squire et al., 2004; Ross, 2007; Moon et al., 2008). Hence, exploring the cultural dimensions of customization brands is pivotal for building consumer-based brand value.

#### Special dimensions

Special dimensions refer to measures only applicable to customization brands. They should reflect the value associated with future sales and profit and focus on self-characteristics that competitors cannot easily duplicate (Aaker, 1996). The dimensions of brand experience and brand services have to be considered according to the characteristics of CACBs.

##### Brand experience

Brand experience is defined as the subjective responses that are evoked in consumers by specific brand-related experience attributes in specified settings (Brakus et al., 2009; Kim, 2012). For example, exquisite store display is visually appealing for consumers, and enthusiastic, thoughtful service lets them feel a type of emotional warm, thereby arousing their subjective response. In the context of the fashion industry, brand experience is composed of cognitive, affective and behavioral aspects (Kim, 2012), all of which enrich the connotation of brand experience dimension. Hence, brand experience is highly related to the CACB.

##### Brand services

Brand services refer to the marketing activities provided by the brand to meet consumers’ needs (Sok and O’Cass, 2011), thus playing an evident role in enhancing brand value (Berry, 2000). Service guarantee, service attitude and service ability as a means to increase service quality are highly related to the customization brand service (Björlin Lidén and Sandén, 2004; Kuo, 2007). In this vein, brand service will influence the brand value of the CACB.

## Development of research questions

### Optimizing a conceptual framework of China apparel customization brands’ brand value

**FIGURE 1:**
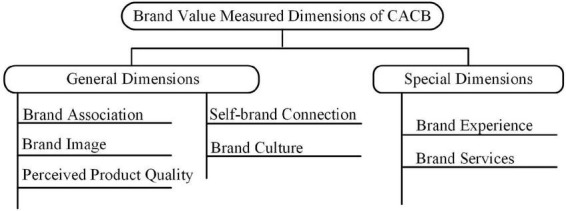
A conceptual framework of CACBs’ brand value measured dimension.

This study developed a proposed conceptual framework of CACBs’ brand value measured dimension (Figure 1) according to existing theoretical studies. The two aspects of brand value are its general dimensions (e.g., brand association, brand image, perceived product quality, self-brand connection, and brand culture) and special dimensions (e.g., brand experience and brand services).

To optimize the conceptual framework of CACBs’ brand value, a semi-structured interview approach was adopted to help practitioners understand the core factors of apparel customization brands. For instance, most practitioners will use market indicators, such as turnover, as a measure of brand value. Hence, the first research question (RQ) of this study is as follows:

**RQ1** Do brand practitioners endorse the measured dimensions in the conceptual framework of brand value?

### Validating the measured dimensions for China apparel customization brands’ brand value

Brand practitioners are the most direct stakeholders of brand value and can have sensitive and accurate perceptions of brand value. However, consumers evaluate the brand value with their own brand knowledge, including brand awareness and brand image (Keller, 1993). If CACBs fail to provide consumers with positive cognitive judgment and affective feelings, consumers are not able to resonate with the brand (Kim, 2012). Brand innovation and perceived product quality in the co-design process likewise affects the brand experience, manifesting in the tailor’s rich textile experience and trendy design ideas (Coelho et al., 2020). Given the differences in the understanding of brand value between practitioners and consumers, the conceptual framework of brand value optimized from practitioners’ perspective requires an empirical test. Thus, the second research question of this study is proposed below:

**RQ2** Do consumers recognize the measured dimensions of brand value optimized from the perspective of brand practitioners?

## Methods and results

### Study 1: Qualitative study – Semi-structured interview

#### Research samples

This study performed purposeful sampling from the brand’s perspective to obtain additional valuable data from non-probabilistic samples that meet certain conditions. The sample requirements were as follows: (1) the sample brands produce clothing using customized methods and models, (2) the customization brand must be in operation for more than 3 years, and (3) the interviewee holds a full-time position in the customization brand. Overall, a total of 11 interviewees participated in this study (Table 1), all of whom have long-term work experience in highly influential brands in the Chinese apparel customization industry, ensuring high-quality insights in the interviews.

**TABLE 1:** Interview sample profile.

No.	Position	Length of service (years)	Company strategic position	Main business income (dollar)^a^	Main product categories
1	Marketing manager	7	Follower	4,500,000	Suit, coat, suit vest women’s suit
2	Co-founder	5	Market leader	18,000,000	Shirts, trousers
3	Marketing manager	6	Market leader	450,000	British shirt, business suit, casual trousers, jacket
4	Creative director	20	Niche leader	–^b^	Chinese traditional gown, cheongsam
5	Salesman	3	Follower	450,000	Cheongsam
6	Marketing director	4	Follower	450,000	Professional attire
7	Marketing director	20	Market leader	525,000	Suit, jacket, shirts
8	Salesman	3	Niche leader	300,000	Cheongsam, Chinese wedding dress
9	Creative director	3	Follower	–*^b^*	Dress, cheongsam
10	Marketing manager	5	Follower	450,000	Shirts, business suit
11	Salesman	3	Follower	525,000	Suit, jacket, shirts

^a^“Main business income (dollar)” refers to the income of the company for which the interviewee works. ^b^“–” indicates that the information is not available.

#### Data collection

This study developed an interview outline for the semi-structured interviews using the following questions: (1) What is brand value? (2) Does the apparel customization brand have brand value? If yes, which aspects or dimensions can be used to measure the customization brand value? If not, why? (3) Can general dimensions (e.g., perceived product quality, brand image, etc.) be used to measure the brand value of CACB? What about its special dimensions? Why? (4) Has your company made corresponding efforts to enhance brand value? How is it done? If not, why? (5) Does the company have any plans to enhance its brand value in the future? If yes, what does this plan look like? If not, why? After the second question, the CACBs’ brand value conceptual framework will be explained to move on to the third question, until the process ends. In each interview, permission to use recording equipment was asked from each interviewee. After the interview, each interviewee was individually numbered. Meanwhile, voice transcription and data analysis were performed immediately. The interview data reached theoretical saturation when no further important information was obtained (Strausss and Corbin, 1990).

#### Coding

**FIGURE 2:**
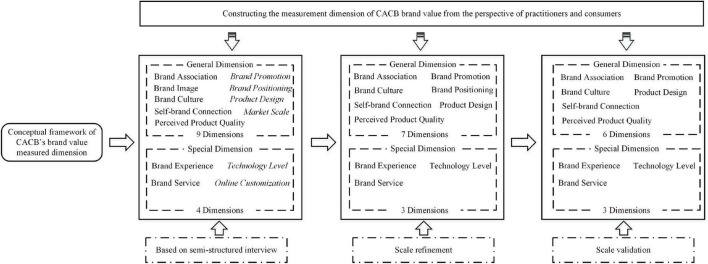
Brand value dimension extraction process.

This research conducted a qualitative analysis and coded the textual data of the interviews by using NVivo 11.0 based on grounded theory and established a theoretical model for brand value (Walker and Myrick, 2006). Open coding, axial coding and selection coding processes were strictly conducted, which extracted 13 brand value dimensions. According to the interview results, four and two new dimensions (in italics) were added into the general and special dimensions respectively, and the brand value dimensions forming process is shown in Figure 2.

### Study 2: Quantitative study – Online survey

#### Initial scale

A 13-dimensional measurement scale containing 84 items was developed based on the brand value conceptual framework of CACBs and the interview-oriented brand value dimensions (Figure 2). To ensure the credibility and structural validity of the generated items, we invited seven experienced researchers (three college apparel brand professional teachers with Doctorate degrees, and four customization brand practitioners with more than 5 years of industry experiences) to perform a card-sorting method on 84 measurement items. They were asked to read through the out-of-sequence items and then put the items into different groups according to the meaning of the items. This process ensured that each group of items could express the same meaning. It also aided the deletion of ‘ambiguous,’ ‘unclear,’ ‘unnecessary,’ and ‘unrepresentative’ items. Based on card sorting results, this research deleted six items, merged two items, and modified seven items. As a result, this study obtained a scale containing 77 measurement items with 13 dimensions (Table 2).

**TABLE 2:** The source and count of initial scale.

Proposed outline	Dimension	Count of item	Source
General dimensions	Brand association	5	(Keller, 1993; Aaker, 1996; Crawford Camiciottoli et al., 2014), interview
	Perceived product quality	4	(Erdoğmuş and Büdeyri-Turan, 2012; Gök et al., 2019; Rosillo-Díaz et al., 2020), interview
	Brand image	5	(Keller, 1993; Cho and Fiore Ann, 2015), interview
	Self-brand connection	5	(Escalas and Bettman, 2003; Ye et al., 2015; van der Westhuizen, 2018; Sarkar et al., 2021), interview
	Brand culture	5	(Lam, 2007), interview
	Brand promotion	6	(Huang and Sarigöllü, 2014; Sandeep and Sidheswar, 2017), interview
	Market scale	4	(Tong and Hawley, 2009; Huang and Sarigöllü, 2014), interview
	Product design	6	(Mishra et al., 2015), interview
	Brand positioning	6	(Bilgili and Ozkul, 2015), interview
Special dimensions	Brand experience	5	(Brakus et al., 2009; Kim, 2012), interview
	Brand service	17	(Björlin Lidén and Sandén, 2004; Kuo, 2007; Sok and O’Cass, 2011), interview
	Technical level	4	(Liu et al., 2020), interview
	Online customization	5	(Zarantonello et al., 2020), interview
*Total*	*15*	*77*	–

#### Scale refinement

A national sample was purchased from Sojump^1^ in June 2021, an online data collection agency. Using judgment sampling, the agency recruited respondents who have customization clothing purchase experience to participate in this study. In the questionnaire, participants were first asked: “Have you ever bought customized clothing?” with the answering process subsequently terminated if the answer was “No” and the answering result would be unaccounted in the number of answer sheets. Otherwise, the respondent would continue answering the questionnaire. As a result, 285 valid questionnaires were extracted. Except for demographic information, all questions used a seven-point Likert scale, ranging from 1 (strongly disagree) to 7 (strongly agree). Before issuing a large-scale questionnaire, a small-scale questionnaire test was conducted to check for clarity and corrections were then made accordingly.

SPSS 20.0 software was used to test the reliability of the questionnaire. The Cronbach’s α of the overall questionnaire was over 0.9, indicating the possibility of unnecessary duplication of content across items (Streiner, 2003). However, the Cronbach’s α of each dimension did not exceed 0.853, indicating the questionnaire’s good reliability.

##### Sample profile

As shown in Table 3, the majority of respondents were female (65.3%). Most of the respondents were aged between 31 and 40 years (44.6%), followed by those aged between 26 and 30 years (35.1%). Approximately 40.7% were managers in enterprises and institutions. A total 75.1% of respondents had a bachelor’s degree, indicating the people with a high degree of education occupied major positions among the sample.

**TABLE 3:** Sample profile.

Demographic factors	Count (*N*)	Percentage (%)
**Gender**		
Female	186	65.3
Male	99	34.7
**Age**		
18–25	48	16.8
26–30	100	35.1
31–40	127	44.6
More than 41	10	3.5
**Education**		
Below undergraduate	12	4.2
undergraduate diploma	214	75.1
Postgraduate diploma and above	59	20.7
*Total*	*285*	*100*

##### Exploratory factor analysis

The corrected item–total correlation was examined for all 77 items. Results showed that 14 items, with a total correlation of less than 0.50, were excluded (Tian et al., 2001), namely, one for product design, two for brand service, one for brand promotion, two for market scale, one for brand positioning, two for brand culture, one for brand image, two for online customization, and two for self-brand connection. Before factor analysis, we checked the validity of the questionnaire. The results showed that the KMO was 0.946, and the significance of the Bartlett sphere test was 0.000, indicating that the questionnaire was suitable for factor analysis. A strong correlation was determined among the variables as well. Next, the principal component analysis method was used to perform exploratory factor analysis and orthogonal rotation analysis on the remaining 63 items to assess the factor loading and dimensionality. Items with low factor loading (<0.40), high cross loading (>0.40) and low communalities (<0.30) were eliminated (Arnold and Reynolds, 2003; Kang and Johnson, 2011). In addition, an item needed to be deleted when the following situations occur: (1) when the number of items in a component was less than or equal to 2; (2) when items expressed different meanings from other items in the same component. As a result, the rotation converged after eight iterations and 35 items were retained while 10 dimensions were extracted (Figure 2), thus explaining 66.074% of the total variance; furthermore, the factor loading ranged from 0.535 to 0.76 (Table 4).

**TABLE 4:** The result of scale refinement.

Proposed outline	Dimensions	Code	Items	FL	α(VE%)
General dimensions	Brand association (ASS)	ASS2	I can recognize X among other competing brands.	0.645	0.766 (4.643)
		ASS3	I know X.	0.621	
		ASS4	Some characteristics quickly come to my mind when X is mentioned.	0.645	
		ASS5	I can quickly recall the symbol or logo of X.	0.713	
	Perceived product quality (QUE)	QUE1	X’s fabric and accessories quality is very good.	0.611	0.778 (3.653)
		QUE2	X’s products are durable.	0.598	
		QUE3	X has very good fabrication tech.	0.577	
		QUE4	X has high quality	0.736	
	Self-brand connection (SBC)	SBC1	Wearing X products can represent my style in fashion.	0.748	0.802 (3.332)
		SBC4	Wearing X products can make a good impression on others.	0.742	
		SBC5	Wearing X products makes me more likely to be recognized by others.	0.738	
	Brand culture (CUL)	CUL2	X’ s historical culture is very attractive to me	0.723	0.764 (3.3)
		CUL3	I really like the X profound cultural foundation by long-term accumulation.	0.72	
		CUL4	X’ s historical heritage (technology, business philosophy, etc.) is very attractive to me.	0.746	
	Brand promotion (PRO)	PRO1	X regularly holds exhibitions/fashion shows.	0.651	0.725 (2.578)
		PRO5	X regularly holds theme activities (including online and offline).	0.741	
		PRO6	I know X through live broadcasts/short videos.	0.705	
	Product design (DES)	DES2	X’s clothing color matching is very good.	0.535	0.78 (2.397)
		DES4	X’s design details are very good.	0.541	
		DES5	X’s pattern (including well-fitting, shape, size) is very good.	0.683	
	Brand positioning (POS)	POS1	X has a clear and consistent brand style.	0.609	0.786 (4.336)
		POS2	X has a very clear user group.	0.674	
		POS3	X has a clear dominant product category.	0.537	
		POS6	X has an appropriate price grade.	0.655	
Special dimensions	Brand experience (EXP)	EXP1	Brand X makes a strong impression on my visual sense or other senses.	0.682	0.79 (33.89)
		EXP2	I find brand X interesting in a sensory way.	0.717	
		EXP3	Brand X appeals to my senses.	0.65	
		EXP4	Brand X induces feelings and sentiments.	0.698	
	Brand service (SER)	SER1	X provides door-to-door and off-site mailing services.	0.624	0.778 (4.993)
		SER2	X delivers on time.	0.76	
		SER5	X completes the promised service in time.	0.647	
		SER6	X does all the services promised to consumers.	0.739	
	Technical level (TEC)	TEC1	X’s products are highly technological.	0.655	0.715 (2.951)
		TEC2	X has advanced measuring methods (three-dimensional scanning, human body photography, etc.).	0.701	
		TEC3	X has virtual fitting technology.	0.624	

VE%, variance explained%; FL, factor loadings.

#### Scale validation

The second round of data collection was conducted to verify the validity of the scale where 35 items were generated from EFA for scale validation. The same data collection method was used as in the scale refinement phase where 864 valid samples were obtained. The age of respondents ranged from 18 to 60, with a mean age of 29.37. The number of female respondents (51.9%) was higher than that of males (48.1%) with most respondents having a bachelor’s degree (70.7%).

Another exploratory factor analysis and orthogonal rotation analysis was conducted on the second round of data using the same criteria as in the scale refinement phase to determine the number of factors. As a result, five items were excluded, which are POS6 and QUE4 (low factor loading), POS1 and POS2 (high cross loading), POS3 (Other items in the POS dimension were dropped). The rotation converged after seven iterations, where 30 items were retained and nine dimensions were extracted (Figure 2), explaining 66.832% of the total variance and the factor loading ranged of 0.599–0.844 (Table 5). All the items had communalities ranging from 0.535 to 0.81, and any additional item deletion could not provide a meaningful explanation for the factor structure and item separation (Kang and Johnson, 2011).

**TABLE 5:** Brand value scale of apparel customization brands.

Proposed outline	Dimensions	Code	Mean	SD	FL	α(VE%)	CR	AVE
General dimensions	ASS	ASS2	5.706	0.954	0.67	0.749 (7.119)	0.797	0.495
		ASS3			0.691			
		ASS4			0.757			
		ASS5			0.694			
	QUE	QUE1	6.12	0.702	0.66	0.666 (2.82)	0.665	0.399
		QUE2			0.599			
		QUE3			0.634			
	SBC	SBC1	5.394	1.172	0.665	0.728 (3.038)	0.731	0.476
		SBC4			0.694			
		SBC5			0.709			
	CUL	CUL2	5.005	1.348	0.828	0.849 (4.696)	0.851	0.657
		CUL3			0.844			
		CUL4			0.757			
	PRO	PRO1	4.72	1.293	0.778	0.771 (3.43)	0.779	0.543
		PRO5			0.808			
		PRO6			0.61			
	DES	DES2	5.659	1.049	0.657	0.692 (3.37)	0.705	0.444
		DES4			0.63			
		DES5			0.709			
Special dimensions	EXP	EXP1	5.2	1.118	0.672	0.773 (4.16)	0.778	0.469
		EXP2			0.694			
		EXP3			0.755			
		EXP4			0.61			
	SER	SER1	5.542	1.037	0.728	0.825 (34.49)	0.827	0.545
		SER2			0.747			
		SER5			0.798			
		SER6			0.675			
	TEC	TEC1	4.88	1.263	0.72	0.8 (3.711)	0.801	0.574
		TEC2			0.766			
		TEC3			0.785			

VE%, variance explained%; FL, factor loadings; SD, standard deviation; CR, composite reliability; AVE, average variance extracted.

Notably, the dimensions of brand promotion, product design, and technical level were added to the scale through interview, with nine items utilized for characterization. These three dimensions, together with their three respective items, were retained during the refinement and validation phase, indicating that consumers and practitioners agree on the usage of these three dimensions to measure brand value. In the consideration of these three dimensions is a must for future studies. Brand promotion is a brand marketing strategy used to persuade consumers to purchase goods and services (Sandeep and Sidheswar, 2017). Brand promotion affects consumers’ perception of the brand and influences their judgment on brand value. Promotion content and channels play a decisive role in brand promotion effects (Raji et al., 2019). Technical level refers to the technology used to design and fabricate customization clothing. Technology (e.g., CAD, body scanning, virtual fitting, etc.) can shorten the customization process, improve customization efficiency, and bring memorable customization experience to customers (Liu et al., 2020), thereby enhancing brand value. Likewise, product design affects consumer judgment of brand value through features such as color, pattern, and details (Mishra et al., 2015). This study therefore measures the CACBs’ brand value from these three aspects.

##### Convergent and discriminant validity

The construct validity of the model can be assessed through both convergent and discriminant validity. Two indicators are used to assess convergent validity (Fornell and Larcker, 1981; Gökçearslan et al., 2016): (1) Factor loadings of all items should be higher than 0.5; and (2) the composite reliability (CR) should be higher than 0.6. As seen from Table 6, the values of factor loadings and CR were within acceptable ranges, indicating that the construct’s convergent validity is sufficient.

**TABLE 6:** Shared variance and AVE.

Dimensions	QUE	DES	TEC	SER	EXP	PRO	CUL	ASS	SBC
QUE	*0.399*								
DES	0.283	*0.444*							
TEC	0.121	0.119	*0.574*						
SER	0.334	0.284	0.218	*0.545*					
EXP	0.227	0.190	0.224	0.349	*0.469*				
PRO	0.133	0.114	0.275	0.193	0.284	*0.543*			
CUL	0.130	0.102	0.201	0.169	0.235	0.278	*0.657*		
ASS	0.238	0.215	0.121	0.348	0.258	0.189	0.192	*0.495*	
SBC	0.186	0.159	0.158	0.306	0.268	0.208	0.162	0.277	*0.476*

Values along the diagonal (in italics) indicate the average variance extracted (AVE).

The discriminant validity of a model can be assessed by comparing the AVE of each dimension with its shared variance (Fornell and Larcker, 1981; Kline, 2015). As seen in Table 6, the AVE value is above the shared variance, indicating sufficient discriminant validity (Fornell and Larcker, 1981).

##### Confirmatory factor analysis

Confirmatory factor analysis (CFA) was conducted to check the structure of the model by using AMOS 22.0. Model fit indices shown that the model provided a goodness fit to the data (chi-square = 794.6, df = 369, chi-square/df = 2.153, comparative fit index = 0.96, incremental fit index = 0.96, normed fit index = 0.929, relative fit index = 0.916, and root mean square error of approximation = 0.037). All the indicators are within the good value range, indicating that these 30 items reasonably represent the nine dimensions of the customization apparel brand value scale.

## Results

This study constructed a brand value measurement scale of 30 items with nine dimensions for CACBs and divided brand value into both general and special dimensions. After checking the validity and structure of the scale, results indicate that the finalized dimensions were reliable and valid. The general dimension has six dimensions, namely brand association (ASS), perceived product quality (QUE), self-brand connection (SBC), brand culture (CUL), brand promotion (PRO), and product design (DES). Perceived product quality had the highest mean (6.12) and the lowest standard deviation (0.702) in the general dimension. Brand association was the highest among the general dimensions, explaining 7.119% of the total variance. Meanwhile, special dimension has three dimensions, namely brand experience (EXP), brand service (SER), and technical level (TEC). Brand service had the highest mean (5.542), the lowest standard deviation (1.037), and the highest variance explained (34.49%).

Moreover, during the dimensions generation process, practitioners and consumers had cognitive conflicts on the dimensions of marketing scale, brand positioning, and online customization. Through qualitative and quantitative analysis, this study eliminated the possible internal differences in the measurement dimensions of brand value to the greatest possible extent.

## Discussion and conclusion

This study established a brand value scale based on the empirical analysis to provide development direction for CACBs in the emergent consumption market. The existing brand value measurement index system is not suitable for service-orientation and digitalization customization brands. Accordingly, a semi-structured interview from the view of CACBs’ practitioners and an empirical survey from the view of consumers were conducted to introduce the brand value variables of CACBs. Academically, this study provides empirical support for the application of brand value in CACBs and tests the relationship of brand value dimensions proposed by practitioners.

The conceptual framework of brand value optimized through semi-structured interviews shows the brand value characteristics of the CACBs. Specifically, after the end of the first study, six new dimensions were added into brand value scale. These dimensions indicated that practitioners emphasize the brand performance in the sales (market scale), brand operation (brand promotion, brand positioning), product (product design, technical level), and marketing channel (online customization). These dimensions are directly related to the profit of the company, which is also in line with Calvo’s research on brand value’ sources (Calvo Dopico and Calvo Porral, 2012). These dimensions are important aspects for enterprises to enhance their brand value and show the service-orientation and digitalization attributes of apparel customization brands in the current market environment.

The brand value scale constructed through the empirical survey shows the general and special dimensions from the view of consumers, providing in-depth views for building the brand value of CACBs. From the aspect of general dimensions, brand association, self-brand connection, perceived product quality, brand culture, brand promotion, and product design dimensions can be used to measure the brand value of CACBs. Specifically, CACBs strengthens the recall of brand symbols or logos increasing brand association (Crawford Camiciottoli et al., 2014), which is crucial for enhancing brand value in the general dimensions. Since brand association accounts for 7.119% of the total variance of explanations, this proportion is the highest in the general dimension. The self-brand connection dimension both effectively reflects consumer needs for self-expression (van der Westhuizen, 2018) and the characteristics of customized brands. Because consumers still value the quality of customized clothing, the mean value of perceived product quality dimension is at a high level in the study of the purification and validation phase.

Brand culture dimension explains 4.696% of the total variance and involves three aspects. Consumer concern about the profound cultural foundation the most, which is affected by the details of the products, the mode of communication with the consumers, and their views on the future. Constant adherence to the cultural foundation allows consumers to better understand brand culture. Historical culture represents the glory of the past and is the brand’s badge. It will also integrates current social culture and becomes a tool to attract consumers and convey brand value. Historical heritage refers to the retained essence during the brand’s long-term development, including clothing fabrication skills and brand business philosophy. Analysis of the constituent elements of historical heritage provides valuable strategies for brand development.

For brand promotion, customization brands convey brand information to consumers by staging both online and offline themed activities, thereby enticing more consumers to purchase products. These activities generally include for-profit themes (e.g., promotional weeks, personal image design courses, and membership clubs) and non-profit ones (e.g., environmental themes, labor themes) and are carried out through online or offline channels depending on their scale and scope. Consumers are willing to learn about brands through live broadcasts and short videos, which customization brands can use as brand promotion channels. Holding exhibitions and fashion shows to display product style characteristics can make consumers better understand the products the brand offer. An item that evaluates clothing pattern has the largest factor loading (0.709) in the product design dimension. Thus, the most important thing for consumers is the clothing pattern, which affects the product style and the comfort. Design details and colors also affect consumers’ judgment of brand value.

From the special dimensions aspect, brand value can be measured using its three dimensions. Virtual fitting technology provides consumers increasingly personalized and customized solutions. Consumers ask for newer requirements in CACBs hinged on the application of new technology. This includes requests such as advanced body measuring methods and high-tech products, which have been neglected in previous brand value measurement scales studies. Furthermore, the scale provides a deeper understanding of brand experience for CACBs’ practitioners, in areas such as optimizing sensory experience (e.g., pictures, video, music, and color) of different retail channels and increasing meaningful brand engagement. Brands induce positive feelings and sentiments, providing emotional resonance with consumers and enabling consumers to have a good brand experience. Brand service is the most important to brand value and explains 34.49% of the total variance. Given the gradual standardization of the service market, consumers value whether the brand can deliver the service promised in a timely manner. Besides, providing convenient door-to-door and off-site mailing services have become the standard configuration of brand services.

Particular, practitioners and consumers have cognitive conflicts on the three dimensions of marketing scale, brand positioning, and online customization, which may affect the reliability of the measurement dimension of brand value. Cognitive conflict reflects differences in judgment among partners about achieving common goals (Chai et al., 2020). This study extracts the views of both parties on brand value through two stages: bridging the possible internal differences in the measurement dimension of brand value to the greatest extent (Cai et al., 2013) and providing a reliable measurement tool for future practitioners. Specifically, post quantitative analysis, the three dimensions extracted from the perspective of practitioner brand value dimensions were removed. Although the marketing scale dimension is widely used to measure brand value, and interviewees have always emphasized brand positioning, these dimensions cannot be directly experienced by consumers and cannot resonate with them (Kim, 2012). Moreover, online customization, as an online sales channel, provides convenience for consumers to customize products, place orders, and inquire. However, practitioners only emphasize its functional attributes but ignore consumer demand for channel experience, making it difficult for consumers to exact a positive emotional relationship with brands through online channels (Huang et al., 2015). Hence, from the consumers’ perspective, the online customization dimension cannot be used as a criterion for evaluating CACBs’ brand value. Therefore, it is necessary to pay more attention to indicators that consumers can directly experience and resonate with them.

## Implications

This study has several important implications. First, based on theoretical analysis of apparel customization brand and brand value, it optimized the conceptual framework of CACBs’ brand value measured dimension, providing empirical support from practitioners’ perspective. Second, by using qualitative and quantitative research methods, a brand value measurement scale of nine dimensions and 30 items was developed for CACBs. Through the division of general dimension and special dimension, this study integrates existing brand value measurement dimensions and the new measurement dimension of CACB. The result makes an important contribution to the CACBs’ brand value measurement structure, providing a solid foundation for future research. Future relevant studies can expand or modify the measurement dimension of customized clothing brand value based on this scale according to new and emerging characteristics of the customized clothing market. Third, for the first time, this study simultaneously investigated practitioners’ and consumers’ perceptions of CACBs’ brand value. This study deepens practitioners’ and consumers’ understanding of brand value by analyzing conflicting measurement dimensions. Results suggest that practitioners should consider validated measured dimensions based on their own perspectives and pay more attention to three dimensions with significant cognitive differences when constructing consumer-based brand value measurement dimensions.

Although this research has crucial implications to the brand value of CACBs, it also presents some limitations. Its first and primary limitation is that the sample is not balanced in variables such as gender and age. It mainly focuses on females, which might lead to the item evaluation leaning toward a female perspective. The respondent ages are also mainly concentrated on those aged 26–40 years, which is also the main consumer group of customized clothing purchases. Gender and age limitations may therefore be unable to fully represent consumers in the entire apparel customization market. Moreover, given that this study focuses on the Chinese apparel customization market, the brand value scale generated may only be suitable in China. Future research will divide the brand value dimensions based on the different types of customization. It will empirically test the relationship between brand value variables and CACBs’ consumption behaviors.

## Data availability statement

The original contributions presented in this study are included in the article/supplementary material, further inquiries can be directed to the corresponding author.

## Ethics statement

Ethical review and approval was not required for the study on human participants in accordance with the local legislation and institutional requirements. Written informed consent from the patients/participants legal guardian/next of kin was not required to participate in this study in accordance with the national legislation and the institutional requirements.

## Author contributions

All authors contributed to the development of the manuscript including the data collection, data analysis, and the writing phase.

## Footnotes

1
www.wjx.cn
