# Erratum: Scale development of apparel customization brand value: From the perspectives of practitioners and consumers

**DOI:** 10.3389/fpsyg.2023.1145298

**Published:** 2023-01-30

**Authors:** Frontiers Production Office

**Affiliations:** Frontiers Media SA, Lausanne, Switzerland

**Keywords:** China apparel customization brands, brand value, customization practitioners, consumers, scale development, brand value dimensions

Due to a production error, the “†” symbol, denoting “These authors have contributed equally to this work,” was attached to authors Hao Li and Li-Wen Gu. It should be attached to authors Hao Li and Xiao-Gang Liu.

The publisher apologizes for this mistake. The original version of this article has been updated.

